# Advancing cardiovascular care—key insights from the Netherlands Heart Journal 2024

**DOI:** 10.1007/s12471-024-01912-5

**Published:** 2024-11-22

**Authors:** Pim van der Harst, Peter Damman, Joris R. de Groot, Maryam Kavousi, Clara E. E. van Ofwegen-Hanekamp, Martin E. W. Hemels

**Affiliations:** 1 Department of Cardiology, Division of Heart and Lungs, University Medical Centre Utrecht, Utrecht, The Netherlands; 2 Department of Cardiology, Radboud University Medical Centre, Nijmegen, The Netherlands; 3 Department of Cardiology, Heart Centre, Amsterdam University Medical Centres/University of Amsterdam, Amsterdam, The Netherlands; 4 Department of Epidemiology, Erasmus MC, University Medical Centre Rotterdam, Rotterdam, The Netherlands; 5 Department of Cardiology, Diakonessenhuis, Utrecht, The Netherlands; 6 Rijnstate Hospital, Arnhem, The Netherlands; 7 Radboud University Medical Center, Nijmegen, The Netherlands

​
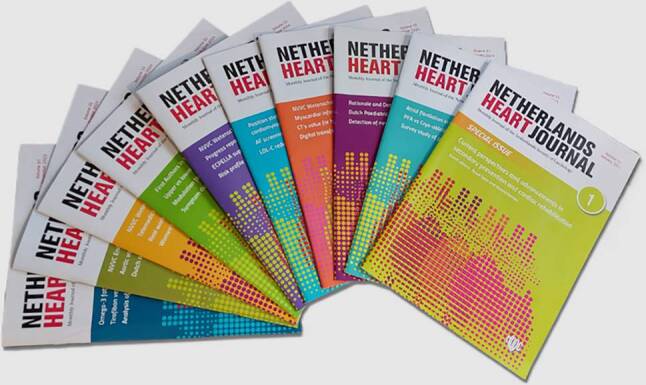


## Introduction

The year 2024 has brought significant innovations in cardiovascular care and research, many of which were highlighted in the *Netherlands Heart Journal*. The journal itself also has undergone further developments, including changes to the format of “Letters to the Editor,” the introduction of the “First Authors in the Spotlight” section, and the formalisation of the process for publishing scientific statements and endorsements from the Netherlands Society of Cardiology (NVVC) [1]. Notably, two special issues were published: one focused on CT imaging for coronary artery disease [2], and the other was dedicated to the hotline sessions and guidelines presented at the European Society of Cardiology congress in London (published only as a hardcopy). All these contribute to consolidating and further expanding on the strong cardiovascular research and care foundation in the Netherlands and beyond through collaboration, discussion and innovation.

In this editorial, we take the opportunity to reflect on some of the most remarkable articles published in *Netherlands Heart Journal* in 2024.

## Innovations in cardiovascular imaging

One of the key themes of this year has been the evolving role of advanced imaging techniques in cardiac diagnostics.

With regards to the use of computed tomography (CT) in coronary artery disease, a noteworthy addition to this year’s progress in diagnostic imaging in the Netherlands is the initiation of the CLEAR-CAD trial, conducted across 30 centres. This trial, led by the Amsterdam University Medical Center and Radboud UMC, is investigating the effectiveness of an upfront CT coronary angiography (CTCA) strategy combined with optimal medical therapy for stable chest pain patients. The trial aims to prevent major adverse cardiac events by using CTCA to guide both medical treatment and selective revascularisation, offering a potentially superior approach to traditional care in managing coronary artery disease [3].

In the acute setting of ST-elevation myocardial infarction, CT scans are often ordered by emergency clinicians to rule out head trauma in out-of-hospital cardiac arrest (OHCA) patients. A study on the use of CT in OHCA patients emphasised both the value and the limitations of this strategy. Findings from the University Medical Center Utrecht suggest that routine head CT before emergency percutaneous coronary intervention (PCI) rarely identifies intracranial haemorrhage. However, it significantly delays PCI, raising the question about the balance between diagnostic thoroughness and timely intervention [4].

In the setting of transcatheter mitral valve replacement (TMVR), 3D computational modelling has been used to improve the procedure. This advanced technique, combining multi-slice CT scans with 3D modelling, allowed clinicians to better plan and guide complex procedures, particularly in cases involving mitral annulus calcification or failed annuloplasty rings. By creating detailed anatomical simulations, researchers from the Erasmus University Medical Center in Rotterdam were able to anticipate and prevent complications such as left ventricular outflow tract obstruction, showcasing the growing role of non-invasive imaging in improving patient outcomes [5].

Researchers from Medisch Spectrum Twente, along with several other Dutch hospitals, further explored the value of routine transthoracic echocardiography (TTE) in stroke management. Their multicentre study, involving patients with ischaemic stroke or transient ischaemic attack of undetermined cause, revealed that TTE detected major cardiac sources of embolism in only 1% of cases. These findings challenge the necessity of routine TTE in these situations, suggesting that its use should be more selective and based on specific clinical indicators [6].

## Developments in cardiovascular pharmacotherapy and interventions

The Amsterdam University Medical Center in collaboration with the Working Group of Cardiology Centres in the Netherlands (WCN) performed a study on the effectiveness of a structured, stepwise approach to lowering LDL‑C in patients after acute coronary syndrome (ACS). The protocol, applied every 4–6 weeks, aimed to reduce LDL‑C to ≤ 1.8 mmol/l, aligning with Dutch guidelines. The structured method helped more patients achieve their LDL‑C targets compared with real-world practices, though keeping those levels low over time remains challenging. This approach highlights the importance, benefits and ongoing difficulties in fully implementing lipid management guidelines after ACS [7].

In aortic valve stenosis management, two studies deserve to be highlighted. First, at the Erasmus University Medical Center in Rotterdam, a study comparing transcatheter aortic valve implantation (TAVI) with surgical aortic valve replacement (SAVR) in patients younger than 75 years found that TAVI was often chosen for higher-risk patients. While TAVI showed higher long-term mortality than SAVR, this was expected given the risk profile of the patients. Importantly, the study reinforced that both treatments are viable options for younger patients, depending on their surgical risk and comorbidities [8]. Optimising future TAVI care, the rationale and design of the TAVI XS trial was published by the Radboud UMC group. In TAVI XS, upper extremity is compared with lower extremity for secondary access (for angiographic guidance and temporary pacing) during TAVI [9].

In electrophysiology, a real-world observational study from the Catharina Hospital in Eindhoven compared two techniques for atrial fibrillation (AF) ablation: cryoballoon ablation and pulsed field ablation (PFA). The study found that PFA resulted in shorter procedure times and fewer complications, such as phrenic nerve palsy, compared with cryoballoon ablation. These findings suggest that PFA may offer a safer and more efficient alternative for pulmonary vein isolation in treating AF [10].

Researchers from Medisch Spectrum Twente explored a novel approach to treating refractory ventricular tachycardia with percutaneous left stellate ganglion block (PSGB). This technique was used when conventional treatments such as medication and catheter ablation were ineffective. The results showed that PSGB could temporarily suppress life-threatening arrhythmias in patients with structural heart disease. While not a permanent solution, it offers a valuable bridge for stabilising patients with electrical storm episodes [11].

## Special populations and needs

This year, several important studies in the *Netherlands Heart Journal* have highlighted the unique needs and outcomes in special cardiovascular populations, such as the geriatric cardiology population, as well as gender-related and socioeconomic considerations.

One study focused on elderly patients with non-ST-elevation myocardial infarction (NSTEMI), drawing data from the nationwide POPular AGE registry. Researchers from St. Antonius Hospital in Nieuwegein, along with other centres, found that ischaemic and bleeding outcomes were high in elderly NSTEMI patients (aged ≥ 75 years). Furthermore, cardiovascular risk was three times higher than bleeding risk in the first year after the NSTEMI. While most elderly patients are treated according to European Society of Cardiology guidelines, the study emphasises the delicate balance between managing bleeding risks and ensuring effective cardiovascular care [12].

In addition to coronary artery disease, elderly patients are at higher risk for ischaemic outcomes due to AF. The multicentre Dutch-GERAF aims to assess the effectiveness and feasibility of an opportunistic screening strategy for clinical AF in frail older patients in 6 centres. Furthermore, observational data will be gathered regarding frailty, the role of biomarkers, and the efficacy and safety of oral anticoagulation [13].

Gender differences in clinical trial participation were examined by the team of the University Medical Center Groningen. This study assessed the inclusion of women in AF trials. They observed that women were well-represented in the Northern Netherlands compared with the general AF population statistics, challenging the global narrative of female underrepresentation in AF trials. Their findings suggest that careful attention to recruitment can help ensure gender equity in clinical research [14].

A group of investigators from Leiden University Medical Centre, Alkmaar Noordwest Ziekenhuisgroep, and Medisch Centrum Leeuwarden combined claims data with area-specific socioeconomic statistics and illustrated treatment patterns and healthcare use in specific regions and patient groups. Their findings emphasise the importance of primary prevention programmes for myocardial infarction patients in low socioeconomic regions as well as development of quality improvement programmes focused on medication adherence after myocardial infarction among inhabitants of high socioeconomic regions [15].

With regards to cardiogenic shock, a study using data from the Netherlands Heart Registry has shown the impact of longer symptom duration and mechanical circulatory support on prognosis in cardiogenic shock complicating acute myocardial infarction [16]. The cardiogenic shock population is complex and known for its subsequent high mortality.

At the University Medical Center Utrecht the combination of extracorporeal membrane oxygenation (ECMO) with Impella, known as ECPELLA, has shown promise for treating patients with refractory cardiogenic shock. This approach allows better oxygen delivery and tissue perfusion in critically ill patients. In this study, 50% of the patients were successfully bridged to long-term solutions such as left ventricular assist devices (LVADs), while 30% recovered fully within 30 days. Despite a high rate of complications, ECPELLA proved to be an effective life-saving option [17].

The same group provided further insights into healthcare consumption for patients receiving an LVAD. This life-saving device is often used in patients with end-stage heart failure. However, the study highlighted the increased need for outpatient visits, emergency care and readmissions over time and underscores the need for more specialised, continuous care for LVAD patients, whose needs often extend beyond the initial implantation [18].

Finally, the Dutch Idiopathic Ventricular Fibrillation (iVF) Registry, the largest of its kind, has made significant contributions in understanding this rare cause of sudden cardiac arrest. By collecting long-term data and data from diagnostic tools such as electrocardiographic imaging with the use of body surface ECGs and a specific heart-torso geometry, the registry aims to uncover hidden triggers of iVF. So far, 9% of the patients initially diagnosed with iVF have been found to have other conditions, and 25% have required appropriate ICD therapy. These findings are helping to refine arrhythmia management and improve patient outcomes [19].

## Advances in telemedicine

This year, the role of telemedicine in cardiovascular care has continued to evolve, especially in the field of heart failure and atrial fibrillation, with several important contributions published in the *Netherlands Heart Journal* from Dutch institutions.

At Maastricht University Medical Center, the TeleCheck-AF project showed how using an app to monitor heart rate and rhythm could reduce the need for in-person consultations and tests such as ECGs. Patients were able to get care remotely without an increase in emergency visits, and most were happy with the new approach. This project has helped pave the way for better integration of remote care into routine AF management [20].

A review article from University Medical Center Utrecht looked at how telemedicine can assist heart failure patients. This review concluded that telemedicine holds promise, but does not work the same for everyone. The research called for generating more real-world data to determine which heart failure patients benefit the most from remote care strategies [21].

## Conclusion

As we reflect on the contributions published in the *Netherlands Heart Journal* in 2024, it is inspiring to see the impressive variety and quality of research that has emerged, particularly from Dutch centres. National registries, innovative clinical trials and expert reviews have driven forward both diagnostic advancements and treatment improvements. The contributions of Dutch research are firmly anchored in the collaborative infrastructure that exists here, supported by organisations including the Netherlands Society of Cardiology (NVVC), the Dutch CardioVascular Alliance (DCVA), the Netherlands Heart Institute (NLHI), and the Working Group on Cardiovascular Research (WCN).

This journal, however, is not only for the Netherlands; it serves the international community. We continue to welcome our international readership and the high-quality research from global contributors, extending our reach to ensure that cardiovascular knowledge and advancements benefit patients everywhere. We are also proud that the *Netherlands Heart Journal* is an open-access journal, allowing unrestricted access to state-of-art research without financial barriers, ensuring that knowledge is shared as widely as possible.

We are grateful to the authors, reviewers and editors who have maintained the high standards of the journal. As we look to 2025, we are confident that, together, we will continue to push the boundaries of cardiovascular knowledge, with impactful research not only from the Netherlands but from around the world.
